# Cost-effectiveness analysis of the Assessment of Burden of Chronic Conditions (ABCC) tool in primary care in the Netherlands

**DOI:** 10.1136/bmjopen-2025-099762

**Published:** 2025-06-24

**Authors:** Loraine HL Peters, Manuela A Joore, Annerika HM Gidding-Slok, Lotte CEM Keijsers, Mascha Twellaar, Esther A Boudewijns, Onno CP van Schayck, Jean WM Muris, Merel L Kimman

**Affiliations:** 1 Department of Family Medicine, Care and Public Health Research Institute (CAPHRI), Maastricht University, Maastricht, The Netherlands; 2 Department of Clinical Epidemiology and Medical Technology Assessment (KEMTA), Maastricht University Medical Centre+ (MUMC+), Maastricht, The Netherlands

**Keywords:** Chronic Disease, Person-Centered Care, Primary Health Care, Self-Management, HEALTH ECONOMICS

## Abstract

**Objectives:**

The increasing prevalence of chronic conditions and multimorbidity places a significant burden on patients and leads to increasing challenges for healthcare systems, especially in primary care. Recognising the multifaceted nature of chronic conditions, the Assessment of Burden of Chronic Conditions (ABCC) tool was developed to support person-centred care, by facilitating shared decision-making and self-management. This study aims to evaluate the cost-effectiveness of the ABCC tool in primary care.

**Design and setting:**

This cost-effectiveness analysis was conducted over 18 months alongside a clustered, two-arm quasi-experimental study in primary care in the Netherlands.

**Participants:**

The study included 231 participants diagnosed with chronic obstructive pulmonary disease (COPD), asthma, type 2 diabetes mellitus (T2DM) and/or chronic heart failure (CHF). Of these, 173 were assigned to the intervention group and 58 to the control group.

**Interventions:**

The intervention group was intended to incorporate the ABCC tool into routine consultations, while the control group had to continue care as usual.

**Outcome measures:**

Outcomes were assessed from a societal perspective, including quality-adjusted life years (QALYs) derived via the EuroQol-5D-5L (EQ-5D-5L) questionnaire. Costs were measured using adapted versions of the Productivity Costs Questionnaire (PCQ) and Medical Consumption Questionnaire (MCQ). Sensitivity analyses (SAs) included a healthcare perspective, per-protocol analysis (to account for disruptions caused by COVID-19) and exclusion of home care costs (to address extreme outliers). Moreover, all analyses were performed for well-being-adjusted life years (WALYs), derived from the ICEpop CAPability measure for Adults (ICECAP-A) questionnaire.

**Results:**

The ABCC tool was more expensive and effective than usual care, with an incremental cost-effectiveness ratio (ICER) of €64 525 per QALY and a 29% probability of cost-effectiveness. With the exception of the healthcare perspective, the SAs yielded more favourable outcomes in terms of cost-effectiveness, with ICERs (probability of cost-effectiveness) of €41 484 (31%), €8683 (58%) and €23 905 (48%) for a healthcare perspective, per-protocol analysis and exclusion of home care costs, respectively. Outcomes for QALY and WALY were comparable.

**Conclusion:**

While the primary analysis suggested a relatively low probability of cost-effectiveness, the SAs showed higher probabilities. The per-protocol analysis suggested that the ABCC tool can be cost-effective when actually used.

**Trial registration number:**

NCT04127383.

**STRENGTHS AND LIMITATIONS OF THIS STUDY**

- The pragmatic design of the trial, with broad inclusion criteria, enhances external validity by closely reflecting real-world clinical settings.
- The pragmatic design does not allow for active promotion of the ABCC tool during the study period.
- The quasi-experimental design without blinding might have introduced possible confounding.

## Introduction

 The rapidly increasing prevalence of chronic conditions has become a major concern for healthcare systems.^1^ In the Netherlands, nearly 60% of the population now lives with at least one chronic condition, a number that has surged by approximately 10% between 2011 and 2022.^2^ This growing trend is expected to continue in the upcoming years, largely driven by the ageing population.^3^ Compounding this issue is the fact that approximately 55% of these cases involve multiple chronic conditions at the same time, a phenomenon known as multimorbidity.^2 3^

The ongoing health problems of chronic conditions, and more specifically multimorbidity, not only burden patients through prolonged suffering and reduced quality of life (QoL), but also put healthcare systems under pressure through increased use of services, resulting in rising healthcare costs.^3^,^5^ In the Netherlands, this challenge is particularly pronounced in primary care, as the majority of care for people with chronic conditions is provided in these settings.^6 7^

The primary challenge lies in effectively delivering appropriate care within these settings. Traditional healthcare systems often face difficulties in meeting the complex long-term care needs of people with chronic conditions.^4 8^ While historically focusing on treating individual diseases rather than addressing holistic patient needs, there is now a shift towards person-centred care.^9^ This change stems from the recognition that traditional approaches can lead to suboptimal health outcomes, raising questions about the adequacy of such systems and highlighting the need for structural changes.

**Figure 1:**
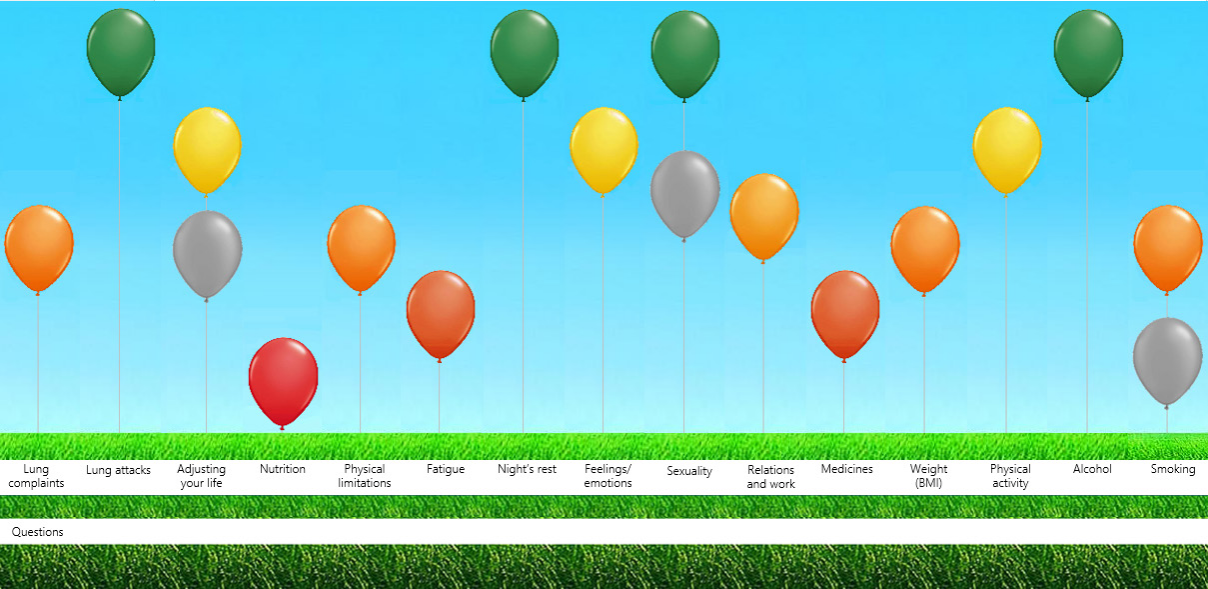
Representation of the ABCC tool for COPD.

In view of the multifaceted nature of chronic conditions, especially in cases of multimorbidity, the Assessment of Burden of Chronic Conditions (ABCC) tool was developed to facilitate more comprehensive and person-centred care in the management of these conditions.^10^ By assessing and visualising the perceived burden of one or more chronic condition(s) in both general and disease-specific domains using a balloon graph (figure 1), this tool can serve as a conversation guide between healthcare provider and patient, promoting shared decision-making and self-management.^10^,^14^

A previously conducted clinical trial demonstrated that using the ABCC tool had a positive impact on perceived quality of care. Additionally, significant improvements were observed in patient activation levels. These outcomes suggest that the ABCC tool may be effective in improving chronic care management.^15^

Besides demonstrating the effectiveness of the ABCC tool, it is crucial to assess the wider implications of its use, especially in terms of cost-effectiveness. Evaluating the cost-effectiveness of a new technology, such as the ABCC tool, compared with standard care is essential to provide a comprehensive understanding of the value of the tool. This economic evaluation provides critical insights for healthcare policymakers, allowing evidence-based decisions to be made on the allocation of scarce healthcare resources, including the possible widespread implementation of the ABCC tool.^16^ Therefore, the current study aims to assess the cost-effectiveness of the ABCC tool in primary care for patients with chronic conditions such as chronic obstructive pulmonary disease (COPD), asthma, type 2 diabetes mellitus (T2DM) and/or chronic heart failure (CHF).

## Methods

### Study design

This trial-based economic evaluation was part of a pragmatic clustered two-armed quasi-experimental study comparing the ABCC tool to usual care, conducted in primary care in the Netherlands. The evaluation was conducted from a societal perspective with an 18-month time horizon. The primary outcome was the quality-adjusted life year (QALY). As one of the objectives of the ABCC tool is to enhance patients’ well-being, beyond health-specific outcomes, the QALY outcomes were complemented by the well-being-adjusted life year (WALY). The trial was developed in 2019 and registered on ClinicalTrials.gov (NCT04127383). Details regarding the study protocol were published elsewhere.^17^ Ethical approval was obtained from the Medical Ethics Committee (METC) of Zuyderland Medical Center, Heerlen, the Netherlands (METCZ20180131). Reporting was done according to the Consolidated Health Economic Evaluation Reporting Standards (CHEERS).^18^

### The Dutch healthcare system

The Dutch healthcare system is structured into primary and specialised (secondary and tertiary) care. All residents are required to have basic health insurance, ensuring access to essential healthcare services. General practitioners (GPs) play a central role and act as gatekeepers to hospital and specialist care, meaning that patients can only access these services through referral.^19 20^

Chronic care in the Netherlands is organised through integrated care programmes, in which various healthcare providers collaborate to offer coordinated care for patients with chronic conditions. This approach is based on care standards that outline protocols for managing chronic conditions like COPD, asthma, T2DM and cardiovascular risk management (CVRM). To support this collaboration, bundled payment models have been introduced, where healthcare providers receive a fixed amount per patient for the entire course of care.^19 21 22^

### Study population, recruitment and allocation

GP practices were recruited for the clinical trial, and allocation to the intervention or control group was determined by the availability of the ABCC tool in each GP practice. Practices with access to the tool were assigned to the intervention group, while those without access constituted the control group. Subsequently, healthcare providers within each practice recruited eligible patients. Patients were eligible for the study if they met the following criteria: a diagnosis of COPD, asthma, T2DM and/or CHF; an age of 18 years or older; and proficiency in understanding and reading the Dutch language. Exclusion criteria were prednisone use for asthma or COPD exacerbations within the 6 weeks prior to the study, as well as hospitalisation for T2DM or CHF patients within the same timeframe.

### Intervention and control groups

GP practices in the intervention group integrated the ABCC tool into their routine consultations (box 1), while practices in the control group continued usual care according to national guidelines, without access to the ABCC tool during the study period.^23^,^26^

Box 1 **Overview of the ABCC tool cycle**

A full description of the ABCC tool, its questions and domains is published elsewhere.^48^ First, before the consultation, patients complete a self-reported questionnaire about their perceived burden of disease (the ABCC scale). Second, the outcomes of the ABCC scale are digitally visualised in a balloon diagram (figure 1). Each balloon symbolises a specific domain of perceived burden, encompassing disease-specific aspects as well as general aspects. Height and colour of the balloons are based on predefined cut-off points and indicate the intensity of the burden experienced by patients. A green balloon signifies a low level of burden, an orange balloon represents a moderate burden and a red balloon indicates a high level of burden. Third, the patient and healthcare provider discuss this graph during the consultation and decide together which domain(s) need(s) specific attention. Clicking on a balloon provides treatment recommendations based on clinical guidelines. Fourth, the patient and healthcare provider together formulate a personalised care plan, including goals, based on the needs and wishes of the patient and available treatment options. In preparation for the next consultation, the cycle begins again with the completion of the questionnaire. This time, balloons from the previous consultation appear in grey, while current outcomes are displayed in colour. These visual distinctions enable easy monitoring of changes in the patient’s experienced burden over time, facilitating ongoing assessment and adjustment of the care plan.^11^

### Measurements

Outcomes were assessed using patient-reported outcome measures (PROMs), which were sent either by e-mail or post. These questionnaires were administered at distinct time points: at baseline (prior to inclusion) and subsequently at 3-month (costs) or 6-month (effects) intervals over a follow-up period of 18 months.

#### Demographic characteristics

Demographic data were collected at baseline using self-reported questionnaires. These included information on age, sex, smoking status, body mass index (BMI), medical history, educational level and national background.

#### Measurement and valuation of effects

The QALYs were based on the EuroQol-5D-5L (EQ-5D-5L), and the WALYs were derived from the ICEpop CAPability measure for Adults (ICECAP-A).^27 28^

The EQ-5D-5L health index is an instrument that assesses five dimensions of health-related quality of life (HRQoL): mobility, self-care, usual activities, pain or discomfort and anxiety or depression. Each dimension is rated on a scale from 1 (no problems) to 5 (extreme problems).^28^ Using tariffs, health states derived from the EQ-5D-5L were converted into utility scores. These utility scores, ranging from −0.446 (worst imaginable health state or death) to 1 (perfect health), quantify the value of a patient’s health state.^29^ Subsequently, by multiplying the utility of a specific health state by the duration spent in that state, the QALYs could be calculated. The resulting overall QALY score represents the number of QALYs gained or lost during the follow-up period.^16 30^

The ICECAP-A instrument was used to assess capability well-being. This instrument measures five key capabilities: stability, attachment, autonomy, achievement and enjoyment, which are crucial to an individual’s well-being. The questionnaire comprises five questions, each corresponding to one of these capabilities. Answer options range from 1 (no capability) to 4 (full capability).^27 31^ To quantify an individual’s overall state, tariff scores were applied.^32^ The resulting utility scores ranged from 0, indicating no capability, to 1, representing full capability. WALYs could then be obtained by multiplying the utility scores by the time spent in that state. The overall WALY score thus reflects the amount of well-being gained or lost over the follow-up period.

Both the EQ-5D-5L and ICECAP-A were administered at four time points: baseline and 6-month, 12-month and 18-month follow-up.

#### Measurement and valuation of costs

This cost-effectiveness analysis was conducted from a societal perspective, incorporating three main categories of costs: healthcare costs, patient and family costs and productivity costs. Healthcare costs included expenses related to primary care, hospital care, home care and consultations with other healthcare providers. These were assessed using an adapted version of the validated Medical Consumption Questionnaire (MCQ), which was developed by the institute for Medical Technology Assessment (iMTA) in the Netherlands.^33^ This adapted MCQ comprised 16 questions concerning the frequency of contacts with healthcare providers.^17^ The recall period for these questions was set at 3 months. Moreover, healthcare costs included intervention costs, which were estimated based on the annual subscription fees of the digital patient environment in which the ABCC tool was provided during the study. These costs were specific to the intervention group and were not applicable to the control group.

Patient and family costs included preventive measures and informal care. Preventive measures encompassed all out-of-pocket expenses for self-care items, while informal care referred to voluntary, unpaid assistance provided by family members or friends in response to health issues.

Productivity costs comprise costs related to productivity losses due to health issues, which include absenteeism from both paid and unpaid work. These productivity losses were measured using an adapted version of the validated Productivity Cost Questionnaire (PCQ), also developed by the iMTA.^34^ This adapted PCQ consisted of questions concerning the impact of disease on an individual’s ability to perform (un)paid work.^17^ The recall period of these questions was set at 4 weeks.

Both the MCQ and PCQ were administered a total of seven times: baseline and 3-month, 6-month, 9-month, 12-month, 15-month and 18-month follow-up. PCQ data observed at the 3-month intervals were assumed to be representative for the preceding months (e.g. productivity loss at 3 months was applied to months 1 and 2).

Cost items were valued using standard cost prices in the Netherlands. Costs were calculated by multiplying the volumes by cost prices per unit.^17^ Unit cost prices for resource use were primarily obtained from the Dutch manual for cost analysis in healthcare research.^35^ Table 1 presents cost prices per unit of resource use. All cost prices were converted to 2024 euros by means of price index numbers.

**Table 1:** Unit prices, expressed in Euros at 2024 values

Cost category	Unit prices in Euro (2024)*†
**Intervention costs**	
Annual subscription	27^1^
**Healthcare costs**	
Primary care	
Regular consultations‡	
Physical consultations	44^2^
Consultations by phone	22^2^
Visitations	66^2^
GP out-of-hours care	
Physical consultations	120^3^
Consultations by phone	35^3^
Visitations	180^3^
Other healthcare providers	
Consultations with paramedical HCPs§	44^2^
Consultations with mental HCPs¶	84^2^
Hospital care	
Outpatient consultations**	119^2^
Emergency care	340^2^
Hospitalisation	624^2^
Ambulance transport	674^2^
Home care	
Domestic help	31^2^
Assistance with personal care	66^2^
Nursing care	95^2^
**Patient and family costs**	
Informal care	19^2^
Prevention††	Various^4^
**Costs due to productivity loss**	
Paid work	
Men	50^2^
Women	41^2^
Unpaid work	19^2^

* When necessary, cost prices were converted to 2024 by means of Dutch consumer price index numbers.

† Source of unit price: 1 Based on annual subscription on SanaCoach from Sananet; 2 Dutch manual for cost prices^35^, 3 Based on publications of the Dutch Healthcare Authority, 4 As reported by participants.

‡ Costs of consultations during office hours with a general practitioner or nurse practitioner.

§ Costs of consultations with a physiotherapist or dietician; for both, the cost of physiotherapy was retained.

¶ Costs of consultations with a psychologist.

** Costs of consultations with a specialist or nurse at a hospital outpatient.

†† Costs incurred by participants to improve their health.

GP, general practice; HCP, healthcare provider.

Costs related to productivity losses were categorised into paid and unpaid work. Absenteeism from paid work due to sick leave was quantified using the friction cost method, which calculates productivity loss costs for the duration of the friction period, i.e. the time needed to replace an employee who is absent due to illness. In the Netherlands, this is calculated to be 143 days (20 weeks).^36^ Thus, costs were calculated over a maximum of 20 weeks in one period of sick leave. Both unpaid work and informal care were valued using the replacement cost method, based on the costs of hiring domestic help.^35^

To account for the time value of costs and effects, costs were discounted by 4% and effects were discounted by 1.5% at T15 and T18.^35^

### Statistical analyses

Data of the base case analysis were analysed according to the modified intention-to-treat (mITT) principle. Patients who died early in the study were excluded because data were not available for these patients and their deaths were not related to the intervention. Missing data on item level (e.g. a patient reported to have received home care or visited a physiotherapist but did not report the number of hours or visits) was resolved by making assumptions based on means per group and other time points. Subsequently, for missing data on the questionnaire level, utilities and cost category data were estimated using multiple imputation by chained equations, with predictive mean matching to account for the skewed distribution of costs.^37 38^ The dataset was split by treatment group (i.e. ABCC tool vs standard care) before imputation, as recommended by Faria *et al*.^39^ Due to the extremely skewed distribution of home care costs, with 95% of the intervention group and 99% of the control group reporting no costs, imputation via multiple imputation was not feasible. To address this, home care costs were imputed separately by assigning the most frequent value within each participant’s time series. The imputation model included patient characteristics (e.g. age, sex, education), clinical characteristics (i.e. chronic conditions) and the outcome variables at other time points. The number of imputations was set at 15 imputed datasets based on the percentage of missing outcome variables. All 15 datasets were analysed separately as described below, and results were pooled using Rubin’s Rules.^40^

The mean differences in costs and effects between the ABCC tool and standard care were estimated using linear regression models, adjusted for baseline differences in costs and utilities and age, sex and clinical conditions.^41^ The incremental cost-effectiveness ratio (ICER) was calculated by dividing the mean difference in costs by the mean difference in effects. To study the uncertainty surrounding the differences in costs and effects, non-parametric bootstrapping with 5000 replications was used and results were plotted on a cost-effectiveness (CE-) plane. The proportion of bootstrapped cost-effect pairs in each quadrant of the CE-plane is also presented. A cost-effect pair located in the northeast quadrant, for example, indicates that the intervention is on average more effective and costly than the comparator, while a cost-effect pair located in the southeast quadrant indicates that the intervention is on average more effective and less costly (dominant) than the comparator. Finally, cost-effectiveness acceptability curves (CEACs) were estimated that show the probability that the ABCC tool is cost-effective in comparison with usual care for different ceiling ratios, that is, the amount of money that society is willing to invest to gain one unit of improvement in a specific effect outcome. For this patient population, the willingness-to-pay threshold was set at €20 000, in line with the guidelines established by the National Health Care Institute in The Netherlands.^42^

All analyses were carried out using R, following recommended methods on trial-based economic evaluations.^43^

### Sensitivity analyses

To assess the robustness of the results, several scenario and sensitivity analyses (SAs) were conducted. First, the cost-effectiveness analysis was performed from a healthcare perspective, meaning that productivity costs and family and patient costs were excluded. Additionally, a per-protocol analysis, excluding participants who did not adhere to the protocol, was performed to evaluate whether deviations from the treatment protocol influenced the outcomes. This analysis was specifically conducted due to the challenges introduced by the COVID-19 pandemic, which disrupted healthcare services and limited interactions with the ABCC tool. As a result, some participants in the intervention group did not use the ABCC tool, which could have biased the results. Therefore, the per-protocol analysis excluded those in the intervention group who did not use the ABCC tool during the study period. Furthermore, a SA was performed in which costs associated with home care were excluded. Notably, home care costs were disproportionately distributed, with patients, all of whom were part of the intervention group, reporting such costs at baseline (ranging from €52 to €6370), while in the control group no patients reported any home care costs. These home care costs continued during the trial and, even with correction for baseline differences, significantly impacted the results. Finally, all analyses were repeated with utilities derived from the ICECAP-A questionnaire. In the absence of established threshold values for WALYs, a ceiling ratio of €20 000, as established for QALYs by the Dutch National Health Care Institute, was also applied to WALYs in this study.^42^

### Patient and public involvement

Patients and/or the public were not engaged in the design, conduct, reporting or dissemination of this research.

## Results

A total of 231 patients participated in this study, across 54 GP practices. Of these, 173 patients were assigned to the intervention group, while 58 patients were allocated to the control group. Baseline characteristics of all participants are displayed in table 2 and online supplemental table 1. At baseline, 35 patients were diagnosed with COPD, 28 with asthma, 193 with T2DM and 29 with CHF. Regarding health and well-being, baseline scores differed by 0.031 and 0.055, respectively, both in favour of the control group.

**Table 2:** Baseline characteristics

	Control group (n=58)	Intervention group (n=173)	p value
Age, years, mean (SD)	63.8 (10.4)	63.0 (9.2)	0.561*
Sex, male, n (%)	40 (69.0)	117 (67.6)	0.850†
Diagnosed with COPD, n (%)	7 (12.1)	28 (16.2)	0.449†
Diagnosed with asthma, n (%)	15 (25.9)	13 (7.5)	<0.001†
Diagnosed with type 2 diabetes, n (%)	44 (75.9)	149 (86.1)	0.068†
Diagnosed with heart failure, n (%)	10 (17.2)	19 (11.0)	0.213†
EQ-5D-5L score, mean (SD)	0.875 (0.1)	0.844 (0.2)	0.223*
ICECAP-A score, mean (SD)	0.899 (0.1)	0.844 (0.1)	0.390*

* Independent sample T-test

† χ2 test

### Effects

After 18 months, the (adjusted) QALY difference was 0.019 in favour of the intervention group (95% CI: −0.020; 0.058). Similar results were observed for the WALY outcome, with the intervention group scoring 0.022 higher (95% CI: −0.0011; 0.056) (table 3).

**Table 3:** Effectiveness outcomes and costs (€) for the control and intervention group (based on imputed dataset (m=15))

Outcomes for the total population at 18 months of treatment			
	Mean (SE)		
	Control (n=58)	Intervention (n=173)	Adjusted mean difference (95% CI)*
**Effects**			
QALY (EQ-5D-5L)	1.283 (0.001)	1.269 (0.001)	0.019 (−0.020; 0.058)
WALY (ICECAP-A)	1.324 (0.001)	1.327 (0.001)	0.022 (−0.011; 0.056)
**Costs**			
Healthcare-related costs	3610 (31)	4779 (34)	874 (−620; 2368)
Intervention costs (fixed)	0	27	n/a
Hospital costs	1271 (16)	1168 (32)	−19 (−684; 645)
GP costs	854 (12)	620 (4)	−187 (−465; 88)
Acute/emergency care	163 (3)	217 (5)	107 (−70; 285)
Allied health professionals	1318 (20)	2265 (13)	334 (−624; 1260)
Home care	3 (0)†	410 (0)†	225 (−137; 586)
Costs for patients and family	689 (22)	524 (14)	−275 (−789; 239)
Informal care costs	387 (20)	356 (13)	−80 (−506; 345)
Out of pocket costs	301 (7)	167 (3)	−180 (−456; 97)
Productivity costs/loss	3238 (49)	2895 (52)	−125 (−1812; 1561)
Total societal costs	7537 (74)	8198 (72)	1213 (−1475; 3902)

* Adjusted for baseline differences, age, sex and presence of chronic condition(s).

† The SE is 0 for home care because the home care variable was imputed before the multiple imputation procedure.

EQ-5D-5L, EuroQol-5D-5L; GP, general practitioner; ICECAP-A, ICEpop CAPability measure for Adults; QALY, quality-adjusted life year; WALY, well-being-adjusted life year .

### Costs

After 18 months of treatment, total healthcar-related costs were €874 higher in the intervention group (95% CI: −620; 2368). Patient and family costs were €275 higher in the control group (95% CI: −789; 239). Productivity costs were similar between the two groups (−2 in the intervention group; 95% CI: −449; 446). Overall, total societal costs were €1213 higher in the intervention group (95% CI: −1475; 3902) (table 3).

### Cost-effectiveness

**Figure 2:**
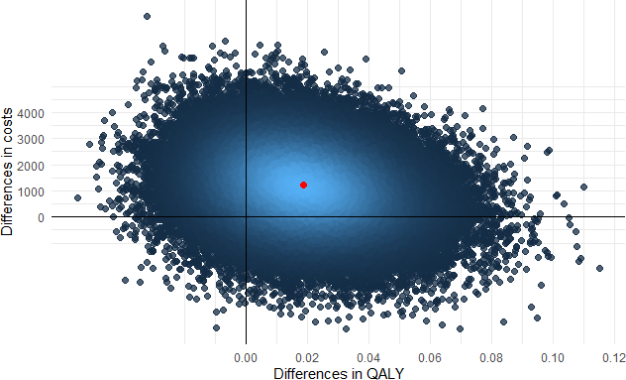
Cost-effectiveness plane representing the joint distribution of the difference in costs and quality-adjusted life years (QALYs) between the control and intervention groups based on 5000 bootstrap simulations, from a societal perspective.

**Figure 3:**
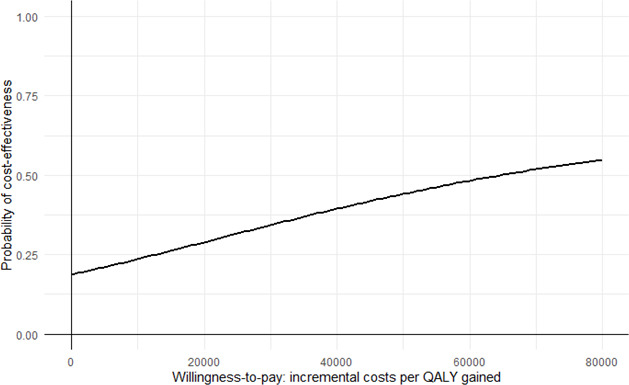
Cost-effectiveness acceptability curve representing the probability that the intervention is cost-effective compared to control for a range of willingness-to-pay thresholds for a QALY, from a societal perspective.

The ICER from a societal perspective was estimated at €64 525 per QALY gained. The majority of bootstrapped cost-effectiveness pairs (65%) were located in the northeast quadrant of the CE-plane, indicating that, on average, the ABCC tool was both more effective and more expensive compared with standard care (table 4, figure 2). The probability that the intervention was considered cost-effective was estimated at 29% (table 4, figure 3).

**Table 4:** Results from the cost-effectiveness analysis (cost per QALY)

Outcome	ΔC (95% CI)*	ΔE (95% CI)*	ICER	CE-plane				Probability of cost-effective intervention at €20 000
				NE (%)	SE (%)	SW (%)	NW (%)	
**Total study population – QALY outcome (EQ-5D-5L)**								
Societal perspective at 18 months (n=231)	1213 (-1475; 3902)	0.019 (−0.020; 0.058)	64 525	65	18	2	15	0.29
Healthcare perspective at 18 months (n=231)	874 (−620; 2368)	0.021 (−0.018; 0.060)	41 484	72	14	2	13	0.31
**Subgroup analysis (societal perspective)**								
Per protocol (n=185)	228 (−2415; 2870)	0.026 (−0.014; 0.066)	8683	50	41	3	6	0.58
**Sensitivity analysis**								
Societal perspective – excl. home care costs (n=231)	463 (−1964; 2891)	0.019 (− 0.020; 0.058)	23 905	53	31	4	12	0.48

* Adjusted for baseline differences, age, sex and presence of chronic condition(s).

CE-plane, cost-effectiveness plane; EQ-5D-5L, EuroQol-5D-5L ; ICER, incremental cost-effectiveness ratio; NE, northeast; NW, northwest; QALY, quality-adjusted life year; SE, southeast; SW, southwest.

From a healthcare perspective, the ICER was found to be €41 484 per QALY, with a 31% probability of cost-effectiveness (table 4).

In the per-protocol analysis, 46 patients were excluded. As a result, the differences in costs decreased to €228 (95% CI: −2415; 2870), while the effect differences increased to 0.026 (95% CI: −0.014; 0.066). This led to an ICER of €8683 per gained QALY. The probability of the intervention being cost-effective increased to 58% (table 4).

The SA, which excluded costs associated with home care, yielded a cost difference of €463 (95% CI: −1964; 2891) and an ICER of €23 905 per additional QALY. The probability of cost-effectiveness was estimated at 48% (table 4).

Cost-effectiveness outcomes for WALY were similar to those for QALY. Detailed results are provided in onlinesupplemental table 2, figures 1  2.

## Discussion

In the current study, a cost-effectiveness analysis was conducted alongside an effectiveness trial, to evaluate the economic implications of the ABCC tool. A total of 231 participants were included, which was lower than the number in the effectiveness study, due to the exclusion of participants who had died during the study period.^15^ From a societal perspective, the primary analysis revealed that €64 525 was required to gain 1 additional QALY. Given that participants in this study had a relatively low disease burden, a willingness-to-pay threshold of 20 000 was applied.^42^ The CEAC showed that the probability of the ABCC tool being cost-effective at this ceiling ratio was only 29%.

The higher costs associated with the intervention mainly came from healthcare-related expenses. These included additional costs for the intervention itself, such as annual subscription costs for the necessary software. However, it is important to note that this software is often already in use within healthcare practices and is not typically purchased solely for the purpose of the intervention. Additionally, patients in the intervention group made greater use of care by allied healthcare professionals. This increase in care is not unexpected, as the ABCC tool helps patients identify challenges posed by their condition and set goals to address these challenges. As a result, they may need additional support from professionals such as physiotherapists, dietitians and occupational therapists to effectively work on these goals. However, over the long term, these costs may decrease as patients become more proactive in managing their health, potentially reducing the need for intensive care, leading to lower healthcare utilisation and associated costs. Finally, patients in the intervention group had higher costs for home care.

Another important thing to note regarding costs is that participants’ medication use was explicitly questioned as part of the study. An in-depth analysis of this data revealed that there were no significant differences between the intervention and control groups. Based on these findings, medication costs were excluded from the economic analysis.

The intervention only had a very small positive effect on health and well-being. This small effect may be explained by the fact that participants already had a relatively high QoL at the start of the study, leaving little room for improvement. Additionally, it is possible that the effects of the intervention were disease-specific and thus not fully captured by general QoL measures, such as the EQ-5D-5L and ICECAP-A questionnaires. Supporting this notion, research on the precursor of the ABCC tool—the Assessment of Burden of COPD (ABC) tool—demonstrated significant improvements in disease-specific QoL, as measured by the COPD-specific St. George’s Respiratory Questionnaire (SGRQ).^44^ Finally, the timeline of this study may not have been long enough to fully capture the long-term benefits of the intervention. It is possible that, over time, as patients continue to use the ABCC tool, the effects may become more pronounced, with cumulative benefits in the management of their chronic conditions.

A deeper exploration of the data was conducted through SAs. First, a per-protocol analysis was performed, as some participants in the intervention group did not use the ABCC tool as intended. In fact, data retrieved from the digital systems used in participating practices indicated that 26.6% of participants in this group did not use the tool at all during the study period. The COVID-19 pandemic likely played a significant role in this. A type 1 effectiveness-implementation hybrid study proved that the implementation of new tools, such as the ABCC tool, is often substantially hindered during crises like this.^45^ Therefore, the per-protocol analysis aimed to better reflect standard conditions, unaffected by the disruptions of a health crisis. Excluding those who had not used the tool resulted in more favourable outcomes, with lower incremental costs per additional QALY of €8683. Consequently, the probability of the intervention being cost-effective increased to 58%. Although it could be argued that patients excluded from the per-protocol analysis may have been in a worse clinical situation because of COVID-19, potentially leading to higher costs and lower effects, this appears unlikely. Patient selection was conducted by healthcare providers, who are assumed to have prioritised patients with relatively stable conditions. This assumption is supported by baseline data, which indicated a generally high QoL among participants. In addition, qualitative research conducted among patients in the intervention group revealed that the majority experienced a relatively low disease burden and consequently perceived limited need to actively engage with the ABCC tool. Nevertheless, participants in this qualitative study acknowledged that the tool would likely be particularly useful for patients with a higher burden of disease (Peters LHL *et al*, unpublished data).

However, it is important to note that while the pandemic may have negatively impacted the use of the ABCC tool during the study, crises like COVID-19 also highlight the potential value of such tools in ensuring the continuity of care. The remote nature of eHealth interventions like the ABCC tool allows for telemonitoring and other forms of digital care. With better preparation and integration into healthcare systems, the tool could play a key role in maintaining care delivery during future crises.

Given the presence of some extreme outliers in home care costs, another SA was performed, excluding these costs. It is worth noting that home care costs in the intervention group were already remarkably high at baseline and therefore probably could not be directly attributed to the intervention. Excluding these costs yielded more favourable outcomes, lowering incremental costs per additional QALY to €23 905 and increasing the probability of the intervention being cost-effective to 48%.

Since chronic conditions not only affect physical health, but also broader aspects of life, this study assessed WALYs in addition to QALYs to provide a more comprehensive assessment and examine whether the two measures provided similar or complementary outcomes. The results showed that QALY and WALY outcomes were comparable. These findings align with those of a recent cost-effectiveness analysis on follow-up care for chronic kidney disease, which used the EQ-5D-5L and ICECAP for older adults (ICECAP-O) to capture QoL. Although this study focused on a different chronic condition and used the ICECAP-O rather than the ICECAP-A, it demonstrated that the choice of questionnaire (i.e. EQ-5D-5L or ICECAP) had minimal impact on the overall cost-effectiveness outcomes.^46^

To our knowledge, this is the first cost-effectiveness assessment of such a tool, and no comparable studies have been done on the cost-effectiveness of similar tools specifically designed to support chronic disease management and person-centred care.

## Strengths and limitations

This study offers several notable strengths. It is the first to assess the cost-effectiveness of a tool designed to measure and visualise perceived disease burden while supporting person-centred care and chronic disease management. The pragmatic design of this trial, with broad inclusion criteria, is a key strength, as it improves external validity by closely mirroring real-world settings. Moreover, the economic evaluation was conducted from a societal perspective, which broadens the applicability of the results. By covering a wide range of costs, including healthcare, patient and family and productivity costs, this study provides a comprehensive picture of the economic impact of the ABCC tool. However, it is important to note that not all costs derived from society could be captured.

Despite these strengths, the study has a number of limitations that need to be acknowledged. First, due to the pragmatic design of the study, the use of the ABCC tool was not actively promoted, which may have led to less frequent use. This limited engagement with the tool may also be attributed to the quasi-experimental design, which may have introduced selection bias, as blinding was not possible. Participants were selected directly by healthcare providers, who may have been hesitant to introduce a new tool to patients with unstable health conditions. Consequently, the sample may be skewed towards individuals with more stable conditions, which is also reflected by the fact that the participants already had a relatively low disease burden at baseline. The COVID-19 pandemic, which occurred during the study period, may have led to such a selection. While the potential selection bias could suggest that the study sample may not be fully representative of the broader patient population, it is expected that this does not compromise the external validity of the findings and that full implementation of the ABCC tool in routine care, especially in patients with a higher disease burden and outside pandemic constraints, could potentially yield more pronounced benefits. In addition, it is important to note that the study was not powered for the EQ-5D-5L and ICECAP-A outcomes, as the sample size was calculated based on the primary outcome of the effectiveness study - the Patient Assessment of Chronic Illness Care (PACIC). Furthermore, the number of participants in the intervention and control groups was unevenly distributed. This imbalance was possibly caused by the COVID-19 pandemic, as recruitment patterns in both groups changed at the start of the pandemic, leading to greater participation in the intervention group. Finally, the study population consisted mainly of patients with T2DM, leaving less evidence for people with COPD, asthma and CHF.

### Future research

The findings of this study are from the Dutch healthcare system, and although the results may be applicable to similar settings, future research should evaluate the (cost-)effectiveness of the ABCC tool in other countries or healthcare systems, both with and without different care delivery models. A similar study will be conducted in South Tyrol, for which the tool will be translated into both German and Italian.^47^

### Conclusion

This study provides valuable insights into the cost-effectiveness of the ABCC tool for chronic disease management within the Dutch healthcare system. The primary analysis showed that the intervention imposes additional costs on society. When these costs are weighed against the effects achieved, the probability of cost-effectiveness seems relatively low. However, SAs reveal more favourable outcomes, with the per-protocol analysis indicating that the probability of cost-effectiveness increases if the ABCC tool is actually used in clinical practice, without disruptions due to health crises such as COVID-19. The results of this study provide important insights for policymakers, healthcare providers, and other relevant stakeholders to guide their decisions regarding the adoption and implementation of the ABCC tool for chronic disease management.

## Supplementary material

10.1136/bmjopen-2025-099762 online supplemental file 1

10.1136/bmjopen-2025-099762 online supplemental file 2

10.1136/bmjopen-2025-099762 online supplemental file 3

## Data Availability

Data are available in a public, open access repository.
