# Supplementary figures and images for: Cost-effectiveness analysis of the Assessment of Burden of Chronic Conditions (ABCC) tool in primary care in the Netherlands
Source: BMJ Open. 2025 Jun 24;15(6):e099762. doi: 10.1136/bmjopen-2025-099762 (PMC12198781; doi:10.1136/bmjopen-2025-099762)

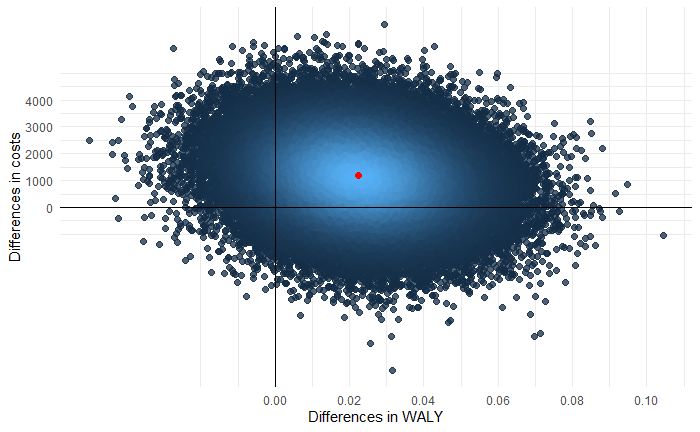

Supplement: online supplemental file 1 [file bmjopen-15-6-s001.tiff]

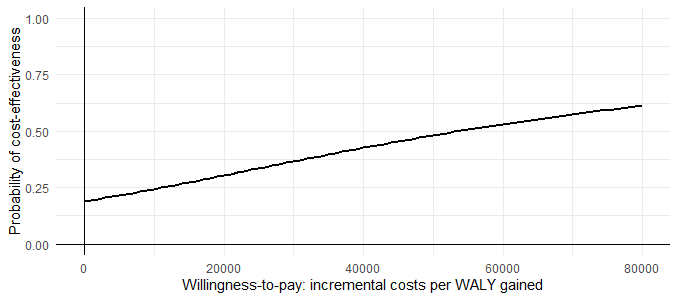

Supplement: online supplemental file 2 [file bmjopen-15-6-s002.tiff]
