# Supplementary material for: Cost-effectiveness analysis of the Assessment of Burden of Chronic Conditions (ABCC) tool in primary care in the Netherlands
Source: BMJ Open. 2025 Jun 24;15(6):e099762. doi: 10.1136/bmjopen-2025-099762 (PMC12198781; doi:10.1136/bmjopen-2025-099762)
Supplement: online supplemental file 3 [file bmjopen-15-6-s003.pdf]

Supplementary data

Supplementary Table 1. Baseline characteristics – additional characteristics

	**Intervention group (n=173)**	**Control group (n=58)**	**p-value**
Age, years, mean (SD)	63.0 (9.2)	63.8 (10.4)	0.561 [1]
Sex, male, n (%)	117 (67.6)	40 (69.0)	0.850 [2]
Smoking status, n (%)			
Never smoked	56 (32.4)	20 (34.5)	0.782 [2]
Ex-smoker	93 (53.8)	32 (55.2)	
Current smoker	24 (13.9)	6 (10.3)	
BMI, kg/m2, mean (SD)	29.8 (5.9)	29.2 (4.5)<br>Missing n=1	0.487 [1]
Diagnosed with COPD	28 (16.2)	7 (12.1)	0.449 [2]
Modified Medical Research Council Dyspnoea questionnaire			
0	0 (0.0)	0 (0.0)	0.659 [3]
1	13 (46.4)	5 (71.4)	
2	11 (39.3)	2 (28.6)	
3	3 (10.7)	0 (0.0)	
4	1 (3.6)	0 (0.0)	
Diagnosed with asthma	13 (7.5)	15 (25.9)	<0.001 [2]
Asthma Control Test; mean (SD)*	18.6 (5.2)	18.7 (5.1)	0.977 [1]
Asthma Control Test			
Not well controlled	4 (30.8)	10 (66.7)	0.128 [4]
Well controlled	9 (69.2)	5 (33.3)	
Diagnosed with type 2 diabetes	149 (86.1)	44 (75.9)	0.068 [2]
Nephropathy	4 (2.7)	4 (9.1)	0.084 [4]
Neuropathy	13 (8.7)	5 (11.4)	0.565 [4]
Eye complications	8 (5.4)	1 (2.3)	0.687 [4]
Sexual complications	29 (19.5)	8 (18.2)	0.850 [2]
Amputation	0 (0.0)	0 (0.0)	NA [5]
Diabetic foot	2 (1.3)	2 (4.5)	0.224 [4]
Cardiovascular disease	31 (20.8)	10 (22.7)	0.784 [2]
Diagnosed with heart failure	19 (11.0)	10 (17.2)	0.213 [2]
NYHA functional classification			
NYHA 1	8 (44.4)	3 (30.0)	0.876 [3]
NYHA 2	6 (33.3)	5 (50.0)	
NYHA 3	4 (22.2)	2 (20.0)	
NYHA 4	0 (0.0)<br>Missing n=1	0 (0.0)	
Educational level, n (%)			
Low	53 (30.8)	26 (44.8)	0.146 [2]
Average	60 (34.9)	17 (29.3)	
High	59 (34.3)<br>Missing n=1	15 (25.9)	
National background, n (%)			
Dutch	149 (86.1)	52 (91.2)	0.676 [3]
1st generation migration	8 (4.6)	1 (1.8)	
2nd generation migration	16 (9.2)	4 (7.0)<br>Missing n=1	

*score ranges from 5 (poor control of asthma) to 25 (complete control of asthma); NYHA = New York Heart Association; [1] Independent sample T-test; [2] Chi square test; [3] Fishers-Freeman-Halton exact test; [4] Fisher's Exact Test; [5] Not applicable

Supplementary Table 2. Results from the cost-effectiveness analysis (cost per WALY)

Outcome	ΔC (95% CI) [a]	ΔE (95% CI) [a]	ICER	CE-plane				Probability of cost-effective intervention at €20.000
				NE (%)	SE (%)	SW (%)	NW (%)	
**Total study population – WALY outcome (ICECAP-A)**								
**Societal perspective at 18 months (n=231)**	637 (-931; 2205)	0.015 (-0.007; 0.037)	42467	69	23	2	7	0.35
**Healthcare perspective at 18 months (n=231)**	741 (-526; 2009)	0.015 (-0.007; 0.037)	49400	77	15	1	7	0.27
**Subgroup analysis (societal perspective)**								
**Per protocol (n=185)**	218 (-682; 1117)	0.018 (-0.005; 0.042)	12111	55	40	2	4	0.57
**Sensitivity analysis**								
**Societal perspective – excl. home care costs**	156 (-1245; 1556)	0.015 (-0.007; 0.037)	10400	52	40	3	5	0.57

[a] Adjusted for baseline differences, age, sex, and presence of chronic condition(s).

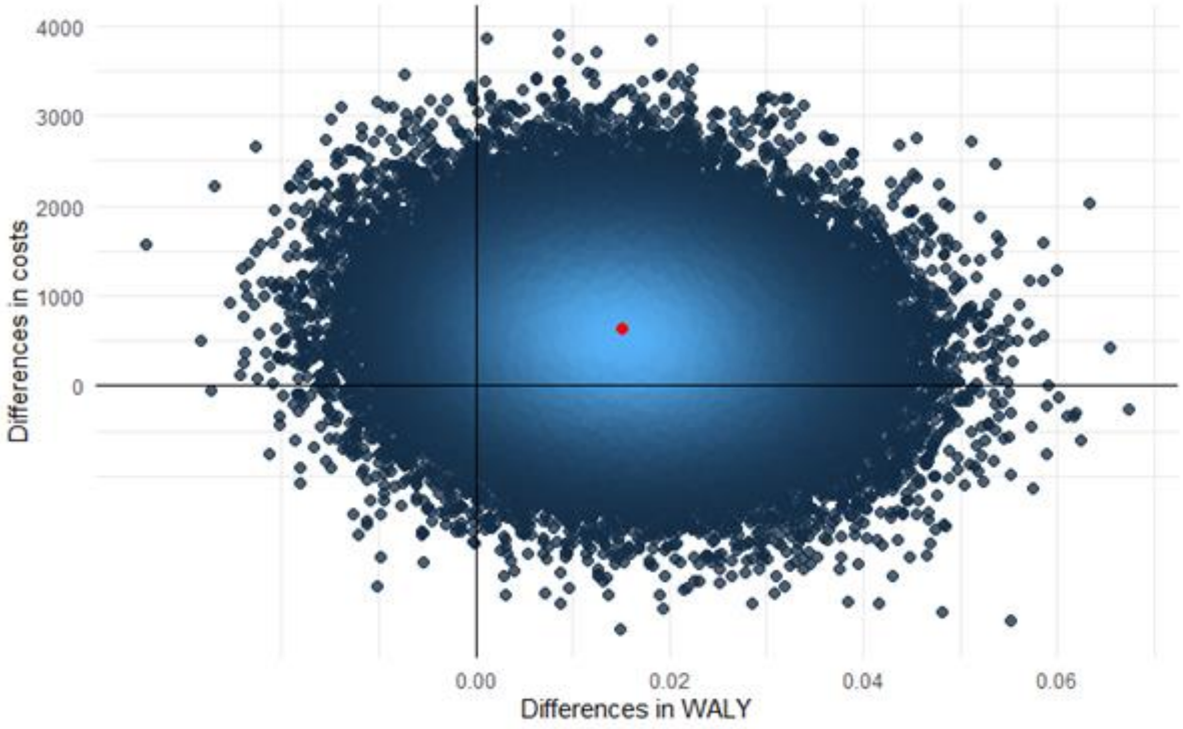

Supplementary Figure 1. Cost-effectiveness plane representing the joint distribution of the difference in costs and WALYs between the control and intervention group based on 5000 bootstrap simulations, from a societal perspective.

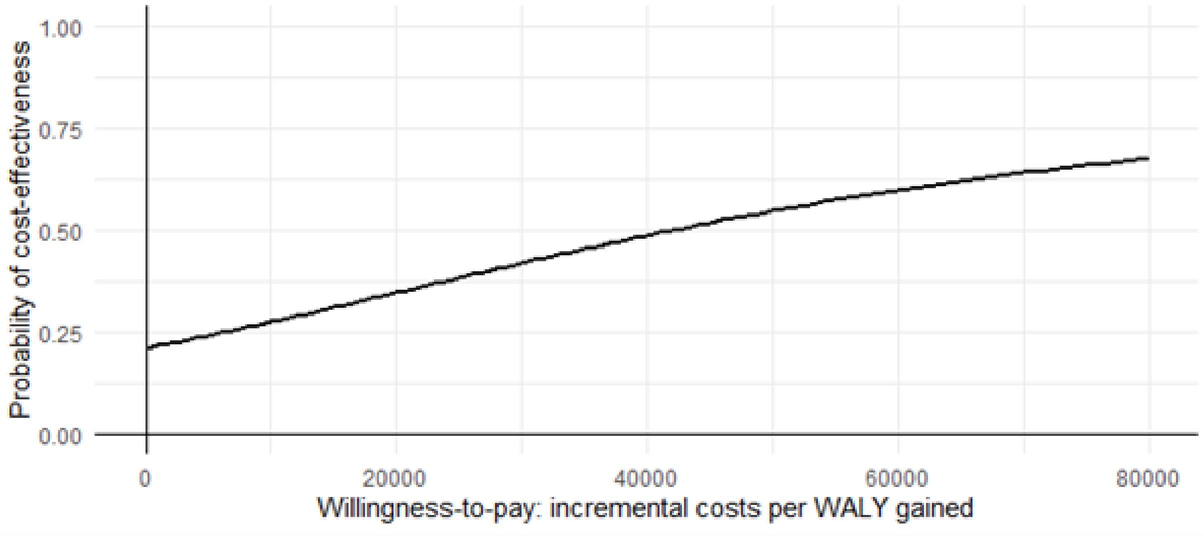

Supplementary Figure 2. Cost-effectiveness acceptability curve representing the probability that the intervention is cost-effective compared to control for a range of willingness-to-pay thresholds for a WALY, from a societal perspective.
