# Supplementary material for: Cost-effectiveness analysis of the Assessment of Burden of Chronic Conditions (ABCC) tool in primary care in the Netherlands
Source: BMJ Open. 2025 Jun 24;15(6):e099762. doi: 10.1136/bmjopen-2025-099762 (PMC12198781; doi:10.1136/bmjopen-2025-099762)
Supplement: Supplementary file 4 [file bmjopen-2025-099762.reviewer_comments.pdf]

# PEER REVIEW HISTORY

BMJ Open publishes all reviews undertaken for accepted manuscripts. Reviewers are asked to complete a checklist review form and are provided with free text boxes to elaborate on their assessment. These free text comments are reproduced below.

## ARTICLE DETAILS

**Title (Provisional)**

Cost-effectiveness analysis of the Assessment of Burden of Chronic Conditions (ABCC) tool in primary care in the Netherlands

**Authors**

Peters, L H L; Joore, Manuela; Gidding-Slok, Annerika HM; Keijsers, L C E M; Twellaar, Mascha; Boudewijns, Esther A.; van Schayck, Onno CP; Muris, Jean WM; Kimman, Merel

---

## VERSION 1 - REVIEW

---

| | |
|---|---|
| **Reviewer** | **1** |
| **Name** | **Aznar-Lou, Ignacio** |
| **Affiliation** | **Sant Joan de Deu Research Institute, PRISMA Research Group** |
| **Date** | **13-Feb-2025** |
| **COI** | **None** |

---

Thank you for giving me the opportunity of reviewing this interesting and well structured paper that assess the efficiency of a tool addressed to manage multimorbidity. Below, you will find some suggestions, mainly focused on the methods section, that in my opinion should be clarified.

Abstract:

Authors pointed that SAs showed more favorable outcomes but it is not in all SAa (healthcare perspective).

Methods

Please, detail when (dates) they trial was developed as well as a brief explanation of the Dutch healthcare system.

Authors used several variables in addition to outcomes and costs, please detail when and how they were obtained.

Please clarify in measurement section if questionnaires were administered monthly for productivity losses as it is specified in “measurement and valuation of costs” when authors pointed that the recall period of these questions is 4 weeks.

I suggest to use more updated unit costs as authors are publishing in 2025 with unit costs from 2021.

Authors did not specify what was the unit cost used to translated paid work to monetary cost, they say they use the friction cost method but they do not report where the data come from. Please, specify it.

Please explain or reference what is the miTT principle.

Taking into account that cost and QALYS does not always have a normal distribution, did authors test other distributions or link fuctions to estimate differences in cost and effects?

How did authors know if patient have used the ABCC tool? Did they have a registry?

In the SA analysis, in the way it is written it seems as societal perspective does not include home care when it has to. Please correct it. Additionally, it would be more accurate say that the perspective used is limited societal as we never be able to registry all cost derived from society.

Authors must justify why they exclude patients who had died during the follow-up.

Results

Please, explain, why home care costs were imputed before the MI process?

In the text, authors say that patient and family costs were higher in the intervention group, however in the table is the opposite. Please, clarify it.

Please include the n in the SA excluding home care costs in Table 4.

Discussion

Those patients excluded in the per-protocol analysis could be in a worse clinical situation because of COVID-19 impacting in higher cost and lower effects. This would only impacted in the intervention group. An explanation about it should be given.

Finally, conflict of interest is not declared. Please include it.

---

| | |
|---|---|
| **Reviewer** | **2** |
| **Name** | **Kelson, Zoe** |
| **Affiliation** | **University of Exeter, Mathematics** |
| **Date** | **07-Mar-2025** |
| **COI** | **None** |

---

This study aims to evaluate the cost-effectiveness of the ABCC tool in primary care.

Reviewer comments:

"This trial-based economic evaluation was part of a pragmatic clustered two-armed quasi-experimental study comparing the ABCC tool to usual care, conducted in primary care in the Netherlands."

and

"The trial was registered on ClinicalTrials.gov (NCT04127383). Details regarding the study protocol were published elsewhere (17)"

Can a copy of the original protocol please be provided for review?

"Reporting was done according to the Consolidated Health Economic Evaluation Reporting Standards (CHEERS)"

Thanks for providing a copy of the CHEERS checklist.

"The study included 231 participants diagnosed with chronic obstructive pulmonary disease (COPD), asthma, type 2 diabetes mellitus (T2DM), and/or chronic heart failure (CHF). Of these, 173 were assigned to the intervention group and 58 to the control group"

Can the authors please further address whether the included sample can be considered to be demographically and characteristically representative?

"allocation to the intervention or control group was determined by the availability of the ABCC tool in each GP practice.

Practices with access to the tool were assigned to the intervention group, while those without access constituted the control group. Subsequently, healthcare providers within each practice recruited eligible patients"

and

"Table 2. Baseline characteristics"

and

"Supplementary Table 1. Baseline characteristics – additional characteristics"

The non-randomised treatment arms appear to be balanced, other than asthma.

Did the authors consider applying propensity score modelling for sample weighting, to adjust for possible confounding differences between treatment groups?

"Missing data on item level (e.g. a patient reported to have received home care or visited a physiotherapist, but did not report the number of hours or visits) was resolved by making assumptions based on means per group and other time points. Subsequently, for missing data on questionnaire level, utilities and cost category data were estimated using multiple imputation by chained equations, with predictive mean matching to account for the skewed

distribution of costs (34, 35). The dataset was split by treatment group (i.e. ABCC tool versus standard care) before imputation, as recommended by Faria et al. (36). The imputation model included patient characteristics (e.g. age, sex, education), clinical characteristics (i.e. chronic conditions) and the outcome variables at other time points. The number of imputations was set at 15 imputed datasets based on the percentage of missing outcome variables. All 15 datasets were analyzed separately as described below and results were pooled using Rubin's Rules (37). "

Missing data has been rigorously handled in the analysis.

Can the authors please clarify how much data was missing for each item, and whether it can be considered to be missing at random?

Can the authors please consider a sensitivity analysis on this approach (e.g. applying a complete case analysis)?

"The mean differences in costs and effects between the ABCC tool and standard care were estimated using linear regression models, adjusted for baseline differences in costs and utilities, and age, sex, and clinical conditions (38). The incremental cost-effectiveness ratio (ICER) was calculated by dividing the mean difference in costs by the mean difference in effects. To study the uncertainty surrounding the differences in costs and effects, non parametric bootstrapping with 5000 replications was used and results were plotted on a cost-effectiveness (CE-) plane. The proportion of bootstrapped cost-effect pairs in each quadrant of the CE-plane is also presented"

and

"Finally, cost effectiveness acceptability curves (CEACs) were estimated that show the probability that the ABCC tool is cost effective in comparison with usual care for different ceiling ratios, i.e. the amount of money that society is willing to invest to gain one unit of improvement in a specific effect outcome. For this patient population, the willingness to-pay threshold was set at €20,000, in line with the guidelines established by the National Health Care Institute in The Netherlands (39)."

The authors have applied appropriate modelling methods, suitably quantifying and communicating uncertainty in the model outputs.

To clarify, does "Adjusted for baseline differences, age, sex, and presence of chronic condition(s)" include asthma as a covariate?

"To assess the robustness of the results, several scenario and sensitivity analyses (SAs) were conducted"

A number of insightful what-if senarios have been explored.

"Due to the pragmatic design, the ABCC tool was not actively promoted, which may have led to lower engagement by users. • The quasi-experimental design introduced the potential for selection bias, as blinding was not feasible, and participants were directly selected by

healthcare providers. • The COVID-19 pandemic hindered the use of the ABCC tool and possibly caused an imbalance in the distribution of participants between the intervention and control groups." [strengths and limitations of this study]

and

"Despite these strengths, the study has a number of limitations that need to be acknowledged. First, due to the pragmatic design of the study, the use of the ABCC tool was not actively promoted, which may have led to less frequent use. This limited engagement with the tool may also be attributed to the quasi-experimental design, which may have introduced selection bias, as blinding was not possible. Participants were selected directly by healthcare providers, who may have been hesitant to introduce a new tool to patients with unstable health conditions. Consequently, the sample may have been skewed towards individuals with more stable conditions, who may have had fewer needs to use the ABCC tool. Moreover, the COVID-19 pandemic, which occurred during the study period, may have further hampered the use of the tool. During that time, healthcare facilities experienced significant disruptions, which affected continuity of care and potentially reduced patient interaction with tools such as the ABCC tool. In addition, it is important to note that the study was not powered for the EQ-5D-5L and ICECAP-A outcomes, as the sample size was calculated based on the primary outcome of the effectiveness study - the Patient Assessment of Chronic Illness Care (PACIC). Furthermore, the number of participants in the intervention and control groups was unevenly distributed. This imbalance was possibly caused by the COVID-19 pandemic, as recruitment patterns in both groups changed at the start of the pandemic, leading to greater participation in the intervention group. Finally, the study population consisted mainly of patients with T2DM, leaving less evidence for people with COPD, asthma, and CHF. "

A discussion on the study limitations is provided.

---

## VERSION 1 - AUTHOR RESPONSE

### Reviewer 1

- Authors pointed that SAs showed more favorable outcomes but it is not in all SAs (healthcare perspective).

  *Changes in the manuscript:*

  ---

  Page 1, line 22-25

  With the exception of the healthcare perspective, the SAs yielded more favorable outcomes in terms of cost-effectiveness, with ICERs (probability of cost-effectiveness) of €41,484 (31%), €8683 (58%),

€23,905 (48%) for a healthcare perspective, per-protocol analysis, and exclusion of home care costs, respectively.

---

- Please, detail when (dates) they trial was developed as well as a brief explanation of the Dutch healthcare system.

*Changes in the manuscript:*

---

Page 6, line 88

The trial was developed in 2019 and registered on ClinicalTrials.gov (NCT04127383).

Page 6, line 92-100

**The Dutch healthcare system**

The Dutch healthcare system is structured into primary and specialized care (secondary and tertiary). All residents are required to have basic health insurance, ensuring access to essential healthcare services. General practitioners (GPs) play a central role and act as gatekeepers to hospital and specialist care, meaning that patients can only access these services through referral.

Chronic care in the Netherlands is organized through integrated care programs, in which various healthcare providers collaborate to offer coordinated care for patients with chronic conditions. This approach is based on care standards that outline protocols for managing chronic conditions like COPD, asthma, T2DM, and cardiovascular risk management (CVRM). To support this collaboration, bundled payment models have been introduced, where healthcare providers receive a fixed amount per patient for the entire course of care.

---

- Authors used several variables in addition to outcomes and costs, please detail when and how they were obtained.

*Changes in the manuscript:*

---

Page 8, line 122-124

***Demographic characteristics***

Demographic data were collected at baseline using self-reported questionnaires. These included information on age, sex, smoking status, body mass index (BMI), medical history, educational level, and national background.

---

- Please clarify in measurement section if questionnaires were administered monthly for productivity losses as it is specified in "measurement and valuation of costs" when authors pointed that the recall period of these questions is 4 weeks.

*Response:* Yes, the iPCQ was administered every 3 months (sent out together with the iMCQ) but the original questionnaire has a standard re-call period of four weeks. It is generally acknowledged that, especially for long trials, it is not always feasible to have continuous data for health care utilization and productivity costs, hence intermittent data collection was used for productivity costs rather than sending the questionnaire every four weeks or adjusting the re-call period. By double-checking the dataset, we found that due to an error, the interpolation technique for the missing months, as recommended in the iPCQ manual and supported by literature (1), had not been applied. We thank the reviewer for paying specific attention to this re-call period through which we discovered this error. The total cost table (Table 3) is now updated with the correct productivity costs. Note that results have slightly changed, as productivity costs were also included in the baseline correction, and multiplying by 3.25 affected the baseline costs (differently) in each group.

For the calculation we assumed that data collected at 3 months, referring to the previous 4 weeks, was representative for months 1-3. Data collected at 6 months was assumed to be representative for months 4-6 and so forth.

*Changes in the manuscript:*

---

Page 15, line 242

Table 3. Effectiveness outcomes and costs (€) for the control and intervention group (based on imputed dataset (m=15))

Page 10, line 163-164

PCQ data observed at the 3-month intervals were assumed to be representative for the preceding months (e.g. productivity loss at 3 months was applied to months 1 and 2).

---

- I suggest to use more updated unit costs as authors are publishing in 2025 with unit costs from 2021.

  *Response*: Initially, we used the year in which most data had been collected. However, we have now updated the prices to 2024 using the Dutch Consumer Price Index. This increased costs for each category (by 17.9%), but did not affect the final results (difference in costs).

  *Changes in the manuscript*:

---

Page 10, line 167-168

All cost prices were converted to 2024 euros by means of price index numbers.

Page 11, line 176

Table 1. Unit prices, expressed in Euros at 2024 values

---

- Authors did not specify what was the unit cost used to translated paid work to monetary cost, they say they use the friction cost method but they do not report where the data come from. Please, specify it.

  *Response*: The unit cost for paid work is presented in Table 1 of the manuscript, but unfortunately there was an error in the source. It said, ‘the friction cost method’, while that is the method, we should have referred to the Dutch manual for cost prices. This has now been corrected.

*Changes in the manuscript:*

---

Page 11, line 176

Table 1. Unit prices, expressed in Euros at 2024 values

---

- Please explain or reference what is the miTT principle.

  *Response*: This means that people who died were not incorporated. Three participants died in the intervention group, already before the first measurement of QoL or costs, and 1 participant died in the control group between 6 and 12 months follow-up. The deaths were not related to the use of the intervention/ABCC tool. As there are no standard methods to analyze the cost (and QoL data) of participants who died, and for three of these patients all data would be missing, we excluded these participants as we thought imputation could have led to unreliable estimates. We do not expect that this affected the outcomes.

  *Changes in the manuscript*:

---

Page 11, line 180-182

Patients who died early in the study were excluded because data were not available for these patients and their deaths were not related to the intervention.

---

- Taking into account that cost and QALYS does not always have a normal distribution, did authors test other distributions or link functions to estimate differences in cost and effects?

  *Response*: Thank you for your insightful comment. We recognize that costs and QALYs often do not follow a normal distribution, and we considered this in our analysis approach. Instead of assuming specific distributions for cost and effect data, we opted for non-parametric bootstrapping, which does not rely on any distributional assumptions and can handle skewed data and potential outliers. It also allows for the

comparison of arithmetic means while accounting for the correlation between costs and effects. This methodology is recommended in the recent tutorial by Ben et al. (2), which highlights its advantages in trial-based economic evaluations, and it was this tutorial (including R code) that we used for this study.

- How did authors know if patient have used the ABCC tool? Did they have a registry?

  *Response*: The ABCC tool has been integrated into digital applications such as the SanaCoach platform developed by Sananet. Through Sananet, we were able to obtain data indicating which patients had used the ABCC tool, as well as information on the frequency and timing of its use.

  *Changes in the manuscript*:

---

Page 20, line 303-304

In fact, data retrieved from the digital systems used in participating practices indicated that 26.6% of participants in this group did not use the tool at all during the study period.

---

- In the SA analysis, in the way it is written it seems as societal perspective does not include home care when it has to. Please correct it. Additionally, it would be more accurate say that the perspective used is limited societal as we never be able to registry all cost derived from society.

  *Changes in the manuscript:*

---

Page 13, line 216-217

Furthermore, a SA was performed in which costs associated with home care were excluded.

Page 21, line 339

However, it is important to note that not all costs derived from society could be captured.

---

- Authors must justify why they exclude patients who had died during the follow-up.

  *Response*: Please see our earlier reply to the question on mITT.

- Please, explain why home care costs were imputed before the MI process

  *Response*: Home care costs could not be imputed using MI due to the extremely skewed distribution. In the intervention group 96-100% of participants did not use home care services, while in the control group this was 99-100%. Since the MI procedure is also split by treatment group, these values (in 99% of cases a value of zero) result in an error in MI. The MI procedure was not possible. Hence, we imputed this variable separately before the MI procedure. This was done by using the most frequently reported value in a participant's time series. As most participants reported zero home care costs at multiple time points, and only a few participants reported some home care costs, we feel this was the most appropriate method as home care costs are often of chronic nature. Another method would have been to add the home care costs to another cost category (e.g. primary care, including GP and allied health professionals) before the imputation process, so that there would not be so many zeros. However, for this study, it was valuable to be able to present GP and allied health professionals use separately and therefore we could not identify a suitable cost category to combine home care costs with.

- In the text, authors say that patient and family costs were higher in the intervention group, however in the table is the opposite. Please clarify it.

  Response: you are right, these costs were higher in the control group

  *Changes in the manuscript:*

  Page 14, line 238-239

Patient and family costs were €275 higher in the control group (95% CI: -789; 239).

---

- Please include the n in the SA excluding home care costs in Table 4.

  *Response*: The n was the same as we only excluded the variable home care costs from these analyses. We have added it to the table for clarification.

  *Changes in the manuscript:*

---

Page 17, line 259

Table 4. Results from the cost-effectiveness analysis (cost per QALY)

n=231

---

- Those patients excluded in the per-protocol analysis could be in a worse clinical situation because of COVID-19 impacting in higher cost and lower effects. This would only impacted in the intervention group. An explanation about it should be given.

  *Response*: We acknowledge that, in theory, patients who were excluded in the per-protocol analysis may have been in a worse clinical situation, which could have led to the ABCC tool not being used. However, we do not believe this was the case. It is expected that during patient selection, healthcare providers primarily included patients they considered to be relatively stable. This is supported by the observation that participants had a relatively high quality of life at baseline. Moreover, a qualitative study (recently submitted for publication (3)) was conducted in which some of the participants in the intervention group of this cost-effectiveness study were interviewed about their experiences with the ABCC tool. The majority of these participants reported experiencing a relatively low disease burden, which limited their personal need to use the ABCC tool. However, they indicated that they believed the ABCC tool would be especially useful for patients with a higher burden of disease.

- Finally, conflict of interest is not declared. Please include it.

  *Changes in the manuscript:*

---

Page 26, line 498-499

***Conflicts of interest***

None declared.

---

**Reviewer 2**

- "This trial-based economic evaluation was part of a pragmatic clustered two-armed quasi-experimental study comparing the ABCC tool to usual care, conducted in primary care in the Netherlands."

  And

  "The trial was registered on ClinicalTrials.gov (NCT04127383). Details regarding the study protocol were published elsewhere (17)"

  Can a copy of the original protocol please be provided for review?

  *Response*: Please see attached a copy of the protocol.

- "Reporting was done according to the Consolidated Health Economic Evaluation Reporting Standards (CHEERS)"

  Thanks for providing a copy of the CHEERS checklist.

  *Response*: A copy is attached to our response.

- "The study included 231 participants diagnosed with chronic obstructive pulmonary disease (COPD), asthma, type 2 diabetes mellitus (T2DM), and/or chronic heart failure (CHF). Of these, 173 were assigned to the intervention group and 58 to the control group"

Can the authors please further address whether the included sample can be considered to be demographically and characteristically representative?

*Response*: Although the participating general practices can be considered fairly typical of Dutch primary care, there may have been some selection bias in the inclusion of patients. Due to the disruptions caused by the COVID-19 pandemic, healthcare providers may have been more inclined to introduce the ABCC tool to patients they considered stable. As a result, the tool was probably mainly used in individuals with a relatively low burden of disease (with thus relatively little room for improvement). While this patient population may not fully representative of the broader population of patients with chronic conditions, we believe that this does not compromise the external validity of the findings, as it is expected that wider application of the ABCC tool , beyond the constraints of the pandemic (with patients who have a higher disease burden), may lead to more pronounced effects.

*Changes in the manuscript*:

---

Page 21, line 244-349

Consequently, the sample may be skewed towards individuals with more stable conditions which is also reflected by the fact that the participants already had a relatively low disease burden at baseline. The COVID-19 pandemic, which occurred during the study period, may have led to such a selection. While the potential selection bias could suggest that the study sample may not be fully representative of the broader patient population, it is expected that this does not compromise the external validity of the findings and that full implementation of the ABCC tool in routine care, especially in patients with a higher disease burden and outside pandemic constraints, could potentially yield more pronounced benefits.

---

- "allocation to the intervention or control group was determined by the availability of the ABCC tool in each GP practice.

Practices with access to the tool were assigned to the intervention group, while those without access constituted the control group. Subsequently, healthcare providers within each practice recruited eligible patients"

and

"Table 2. Baseline characteristics"

And

"Supplementary Table 1. Baseline characteristics – additional characteristics" The non-randomised treatment arms appear to be balanced, other than asthma.

- Did the authors consider applying propensity score modelling for sample weighting, to adjust for possible confounding differences between treatment groups?

  *Response*: Propensity score modeling is indeed a sophisticated method for addressing confounding and is relatively new in the field of trial-based economic evaluations. Traditionally, most trial-based economic evaluations have been conducted alongside (large) RCTs, where randomization helps to minimize confounding. In this study, we chose to account for potential confounders directly in the regression analyses instead, as recommended by Ben at al (2) in their tutorial for economic evaluations. We recognize that this is a simpler and more pragmatic approach, but we believe it was appropriate for the purpose of this study. However, in future studies it would be interesting to investigate propensity score modelling in a non-randomized and unequal study like this and to examine its appropriateness in a population with a fairly large difference in the intervention and control groups (58 vs 173 in this study).

- "Missing data on item level (e.g. a patient reported to have received home care or visited a physiotherapist, but did not report the number of hours or visits) was resolved by

making assumptions based on means per group and other time points. Subsequently, for missing data on questionnaire level, utilities and cost category data were estimated using multiple imputation by chained equations, with predictive mean matching to account for the skewed distribution of costs (34, 35). The dataset was split by treatment group (i.e. ABCC tool versus standard care) before imputation, as recommended by Faria et al. (36). The imputation model included patient characteristics (e.g. age, sex, education), clinical characteristics (i.e. chronic conditions) and the outcome variables at other time points. The number of imputations was set at 15 imputed datasets based on the percentage of missing outcome variables. All 15 datasets were analyzed separately as described below and results were pooled using Rubin's Rules (37)."

Missing data has been rigorously handled in the analysis.

*Response*: Thank you.

- Can the authors please clarify how much data was missing for each item, and whether it can be considered to be missing at random?

*Response*: Please see below the table we have created for you, detailing the missing data.

Missings per cost and effect variable

| | Missings T0 n (%) | Missings T3 n (%) | Missings T6 n (%) | Missings T9 n (%) | Missings T12 n (%) | Missings T15 n (%) | Missings T18 n (%) |
|---|---|---|---|---|---|---|---|
| GP care | | | | | | | |
| Total population | 11 | 26 | 33 | 42 | 51 | 74 | 95 |
| *Intervention* | *9 (5.20)* | *21 (12.14)* | *25 (14.45)* | *33 (19.08)* | *43 (24.86)* | *65 (37.57)* | *81 (46.82)* |
| *Control* | *2 (3.45)* | *5 (8.62)* | *8 (13.79)* | *9 (15.52)* | *8 (13.79)* | *9 (15.52)* | *14 (24.14)* |
| hospital care | | | | | | | |
| Total population | 15 | 24 | 30 | 41 | 50 | 76 | 97 |
| *Intervention* | *11(6.36)* | *18 (10.40)* | *25 (14.45)* | *32 (18.50)* | *42 (24.28)* | *67 (38.73)* | *82 (47.40)* |
| *Control* | *4 (6.90)* | *6 (10.34)* | *5 (8.62)* | *9 (15.52)* | *8 (13.79)* | *9 (15.52)* | *15 (25.86)* |
| acute care | | | | | | | |
| Total population | 4 | 24 | 29 | 40 | 50 | 74 | 94 |
| *Intervention* | *3 (1.73)* | *19 (10.98)* | *24 (13.87)* | *31 (17.92)* | *42 (24.28)* | *66 (38.15)* | *80 (46.24)* |
| *Control* | *1 (1.72)* | *5 (8.62)* | *5 (8.62)* | *9 (15.52)* | *8 (13.79)* | *8 (13.79)* | *14 (24.14)* |
| paramedical care | | | | | | | |
| Total population | 3 | 24 | 29 | 38 | 49 | 73 | 95 |
| *Intervention* | *2 (1.16)* | *19 (10.98)* | *24 (13.87)* | *30 17.34)* | *41 (23.70)* | *65 37.57)* | *81 (46.82)* |

| | | | | | | | |
|---|---|---|---|---|---|---|---|
| *Control group* | *1 (1.72)* | *5 (8.62)* | *5 (8.62)* | *8 (13.79)* | *8 (13.79)* | *8 (13.79)* | *14 (24.14)* |
| Prevention | | | | | | | |
| Total population | 11 | 22 | 31 | 39 | 52 | 74 | 95 |
| *Intervention* | *9 (5.20)* | *17 (9.83)* | *25 (14.45)* | *31 (17.92)* | *43 (24.86)* | *65 (37.57)* | *81 (46.82)* |
| *Control* | *2 (3.45)* | *5 (8.62)* | *5 (8.62)* | *8 (13.79)* | *9 (15.52)* | *9 (15.52)* | *14 (24.14)* |
| informal care | | | | | | | |
| Total population | 4 | 22 | 29 | 38 | 49 | 73 | 94 |
| *Intervention* | *3 (1.73)* | *17 (9.83)* | *24 13.87)* | *30 (17.34)* | *41 (23.70)* | *65 (37.57)* | *80 (46.24)* |
| *Control* | *1 (1.72)* | *5 (8.62)* | *5 (8.62)* | *8 (13.79)* | *8 (13.79)* | *8 (13.79)* | *14 (24.14)* |
| productivity short | | | | | | | |
| Total population | 11 | 26 | 34 | 40 | 52 | 77 | 98 |
| *Intervention* | *9 (5.20)* | *20 (11.56)* | *28 (16.18)* | *32 (18.50)* | *43 (24.86)* | *69 (29.88)* | *84 (48.55)* |
| *Control* | *2 (3.45)* | *6 (10.34)* | *6 (10.34)* | *8 (13.79)* | *9 (15.52)* | *8 (13.79)* | *14 (24.14)* |
| EQ-5D-5L | | | | | | | |
| Total population | 3 | - | 28 | - | 49 | - | 97 |
| *Intervention* | *3 (1.73)* | - | *23 (13.29)* | - | *41 (23.70)* | - | *82 (47.40)* |
| *Control* | *0 (0)* | - | *5 (8.62)* | - | *8 (13.79)* | - | *15 (25.86)* |
| ICECAP-A | | | | | | | |
| Total population | 1 | - | 30 | - | 51 | - | 98 |
| *Intervention* | *1 (0.58)* | - | *25 (14.45)* | - | *42 (24.28)* | - | *84 (48.55)* |
| *Control* | *0 (0)* | - | *5 (8.62)* | - | *9 (15.52)* | - | *14 (24.14)* |

While it is not possible to directly test whether the data are missing at random (MAR), we consider the MAR assumption to be plausible. Since this study collected a wide range of variables—including clinical and demographic characteristics, as well as multiple measurements of both costs and health outcomes—there are likely to be variables that predict the missingness. These were included in the multiple imputation (MI) analysis. MI is the most commonly used and recommended method for handling missing data in trial-based economic evaluations (2, 4-6).

- Can the authors please consider a sensitivity analysis on this approach (e.g. applying a complete case analysis)?

  *Response*: We identified the complete cases, and this was 30 in the control group and 57 in the intervention group. These low numbers (e.g. only 33% of the intervention group) are due to the long follow-up with 3-monthly measurements. We feel that this sample is fairly small for the results to have additional value. Yet, we have added the results for your review (it confirms that the intervention is not cost-effective).

| Outcome | ΔC (95% CI) | ΔE (95% CI) [a] | ICER | CE-plane | | | | Probability of cost-effective intervention at €20.000 |
|---|---|---|---|---|---|---|---|---|
| | | | | NE (%) | SE (%) | SW (%) | NW (%) | |
| | | | | | | | | |
| Complete case analyses (n=87) | 2328 (-2108; 6765) | 0.001 (-0.039; 0.042) | 1647832 | 41 | 11 | 6 | 42 | 0.17 |

- "The mean differences in costs and effects between the ABCC tool and standard care were estimated using linear regression models, adjusted for baseline differences in costs and utilities, and age, sex, and clinical conditions (38). The incremental cost-effectiveness ratio (ICER) was calculated by dividing the mean difference in costs by the mean difference in effects. To study the uncertainty surrounding the differences in costs and effects, non-parametric bootstrapping with 5000 replications was used and results were plotted on a cost-effectiveness (CE-) plane. The proportion of bootstrapped cost-effect pairs in each quadrant of the CE-plane is also presented"

  And

  "Finally, cost effectiveness acceptability curves (CEACs) were estimated that show the probability that the ABCC tool is cost effective in comparison with usual care for different ceiling ratios, i.e. the amount of money that society is willing to invest to gain one unit of improvement in a specific effect outcome. For this patient population, the willingness to-pay threshold was set at €20,000, in line with the guidelines established by the National Health Care Institute in The Netherlands (39)."

  The authors have applied appropriate modelling methods, suitably quantifying and communicating uncertainty in the model outputs.

  *Response*: Thank you.

- To clarify, does "Adjusted for baseline differences, age, sex, and presence of chronic condition(s)" include asthma as a covariate?

  *Response*: Yes, all chronic conditions were separately added to the regression.

- "To assess the robustness of the results, several scenario and sensitivity analyses (SAs) were conducted"

  A number of insightful what-if scenarios have been explored.

  *Response*: Thank you.

**Additional remarks**

We would like to once again thank the reviewers for their valuable critique and feedback, which we have hopefully addressed to their satisfaction. We would like to make a final note regarding an issue that was identified shortly after our initial submission, prior to receiving the decision. We discovered that the QALY gain was incorrectly calculated in the original analysis. Specifically, we had presented the mean QALY gain over one year, whereas it should have been calculated over 18 months.

To be fully transparent, the original (incorrect) formula used was:

```
QALY <- 1/2 * ((E_T0 + E_T6) * 6/18 + (E_T6 + E_T12) * 6/18 + (E_T12 + E_T18) * 6/18)
```

However, the correct formula should have been:

```
QALY <- 1/2 * ((E_T0 + E_T6) * 6/12 + (E_T6 + E_T12) * 6/12 + (E_T12 + E_T18) * 6/12)
```

This calculation has now been applied in the current version of the manuscript. This led to a minor increase in QALYs gained, but did not change our main findings.

**References**

1. Hendriks MR, Al MJ, Bleijlevens MH, van Haastregt JC, Crebolder HF, van Eijk JT, et al. Continuous versus intermittent data collection of health care utilization. Med Decis Making. 2013;33(8):998-1008.
2. Ben Â J, van Dongen JM, El Alili M, Esser JL, Broulíková HM, Bosmans JE. Conducting Trial-Based Economic Evaluations Using R: A Tutorial. Pharmacoeconomics. 2023;41(11):1403-13.

3. Peters LHL, Keijsers LCEM, Schayck OCPv, Muris JWM, Vervloet M, Gidding-Slok AHM. Patient Experiences with the Assessment of Burden of Chronic Conditions (ABCC) Tool in Primary Care: A Qualitative Study (submitted).
4. Burton A, Billingham LJ, Bryan S. Cost-effectiveness in clinical trials: using multiple imputation to deal with incomplete cost data. Clin Trials. 2007;4(2):154-61.
5. El Alili M, van Dongen JM, Esser JL, Heymans MW, van Tulder MW, Bosmans JE. A scoping review of statistical methods for trial-based economic evaluations: The current state of play. Health Econ. 2022;31(12):2680-99.
6. MacNeil Vroomen J, Eekhout I, Dijkgraaf MG, van Hout H, de Rooij SE, Heymans MW, et al. Multiple imputation strategies for zero-inflated cost data in economic evaluations: which method works best? Eur J Health Econ. 2016;17(8):939-50.

## VERSION 2 - REVIEW

| | |
|---|---|
| **Reviewer** | **1** |
| **Name** | **Aznar-Lou, Ignacio** |
| **Affiliation** | **Sant Joan de Deu Research Institute, PRISMA Research Group** |
| **Date** | **14-May-2025** |
| **COI** | |

Thank you for addressing my suggestions.

I am fine with all the modifications done by the authors, however I have just two comments. Both were, in my opinion, correctly addressed in the letter of response but the justification why authors take these decissions is not included in the paper. Please, see below:

Although I agree with the way authors have imputed home care costs, I think an explanation about it should be placed in the manuscript. A summary of the explanations authors gave in their lettet would be fine in the main body of the manuscript.

Similarly, I would suggest to include in the paper the justification why authors think per-protocol patietns will not be in a worse clinical situation as they did in their reponse letter.

## VERSION 2 - AUTHOR RESPONSE

Reviewer 1

1. Although I agree with the way authors have imputed home care costs, I think an explanation about it should be placed in the manuscript. A summary of the explanations authors gave in their letter would be fine in the main body of the manuscript.

Changes in the manuscript:

Page 11, line 178-181

Due to the extremely skewed distribution of home care costs, with 95% of the intervention group and 99% of the control group reporting no costs, imputation via multiple imputation was not feasible. To address this, home care costs were imputed separately by assigning the most frequent value within each participant's time series.

2. Similarly, I would suggest to include in the paper the justification why authors think per-protocol patients will not be in a worse clinical situation as they did in their response letter.

Changes in the manuscript:

Page 18, line 301-309

Although it could be argued that patients excluded from the per-protocol analysis may have been in a worse clinical situation because of COVID-19, potentially leading to higher costs and lower effects, this appears unlikely. Patient selection was conducted by healthcare providers, who are assumed to have prioritized patients with relatively stable conditions. This assumption is supported by baseline data, which indicated a generally high QoL among participants. In addition, qualitative research conducted among patients in the intervention group revealed that the majority experienced a relatively low disease burden and consequently perceived limited need to actively engage with the ABCC tool. Nevertheless, participants in this qualitative study acknowledged that the tool would likely be particularly useful for patients with a higher burden of disease [47].
